# Perfluoroalkane Acids and Fetal Growth

**DOI:** 10.1289/ehp.11036

**Published:** 2008-06

**Authors:** Anthony R. Scialli

**Affiliations:** Sciences International, Inc., Alexandria, Virginia, E-mail: ascialli@sciences.com

In the November issue of *Environmental Health Perspectives*, Apelberg et al. (2007) reported an inverse relationship between umbilical cord blood concentrations of perfluorooctanoate (PFOA) and perfluorooctane sulfonate (PFOS) and ponderal index and head circumference in children delivered vaginally in Baltimore, Maryland. In the same issue, Fei et al. (2007) reported an inverse relationship between first trimester maternal blood PFOA (but not PFOS) concentration and birth weight in Danish infants born to normal-weight women. Although these studies do not necessarily support one another (Fei et al. also collected cord blood but did not report these results), they raise the important question of whether low-level exposure to perfluoroalkane acids might affect fetal growth. In both articles, the authors called attention to the inconsistency between these findings and those in experimental animal studies, in which fetal growth effects occur only at blood concentrations several orders of magnitude higher than were measured in human umbilical cord or maternal blood. The question was reasonably posed by both groups whether a confounder could be responsible for the observed associations. The Baltimore group (Apelberg et al. 2007) identified two candidate confounders that may explain their findings: diet and plasma volume.

Perfluoroalkanesulfonamides, which may be metabolized to PFOS, have been used in grease- and water-repellant packaging for foods, particularly pizza, french fries, and other fried foods. The Canadian Total Diet Study (Tittlemier et al. 2006) detected perfluoroalkanesulfonamides in all foods tested, but the highest concentrations were found in pizza, microwave popcorn, egg breakfast sandwiches, french fries, chicken nuggets, and fish burgers. Fluorotelomer alcohols, which can be converted to the corresponding alkane acids, have been used in coatings for paper, including microwave popcorn bags. 8-2 Fluorotelomer alcohol can be converted atmospherically and metabolically to PFOA, and gavage treatment of pregnant mice with 8-2 fluorotelomer alcohol results in the appearance of PFOA in fetuses (Henderson and Smith 2007). Both 8-2 fluorotelomer alcohol and PFOA have been found in popcorn bags and in the vapor produced after cooking microwave popcorn (Begley et al. 2005; Sinclair et al. 2007).

The pregnancies studied by Fei et al. (2007) occurred in 1996–2002, a period during which perfluorinated compounds were commonly used in fast-food packaging. The use of perfluorinated compounds in food packaging decreased some years before 2004–2005, the study period of Apelberg et al. (2007); however, PFOS and PFOA have long half-lives and may still have been present as markers of a high intake of fast-food. A high intake of fast food may in turn be a marker of poor nutrition. The Danish National Birth Cohort (Fei et al. 2007) included a food frequency questionnaire. It would be interesting to know if a relationship between nutrition and maternal blood perfluoroalkane acid concentration was detected.

PFOA and PFOS repel fat and are distributed in body water, particularly plasma. Women with a reduced plasma or body water volumes would distribute the same body burden of perfluoroalkane acids in a smaller space, producing higher perfluoroalkane acid concentrations. Fat-free body mass and total body water volumes are important predictors of birth weight (Butte et al. 2003; Lederman et al. 1999; Mardones-Santander et al. 1998; Sanin Aguirre et al. 2004), giving rise to the possibility that higher maternal blood (and therefore fetal blood) concentrations of PFOS and PFOA are markers of reduced plasma or total body water volumes, producing an apparent inverse association between the perfluoroalkane acid concentrations and fetal growth.

A reasonable next step in addressing the question of whether perfluoroalkane acids (at current human blood concentrations) play a role in fetal growth will be studies in which maternal nutrition and body composition, as opposed to body weight, are considered as possible confounders.

## References

[b1-ehp0116-a0238a] Apelberg BJ, Witter FR, Herbstman JB, Calafat AM, Halden RU, Needham LL (2007). Cord serum concentrations of perfluorooctane sulfonate (PFOS) and perfluorooctanoate (PFOA) in relation to weight and size at birth. Environ Health Perspect.

[b2-ehp0116-a0238a] Begley TH, White K, Honigfort P, Twaroski ML, Neches R, Walker RA (2005). Perfluorochemicals: potential sources of and migration from food packaging. Food Addit Contam.

[b3-ehp0116-a0238a] Butte NF, Ellis KJ, Wong WW, Hopkinson JM, Smith EO (2003). Composition of gestational weight gain impacts maternal fat retention and infant birth weight. Am J Obstet Gynecol.

[b4-ehp0116-a0238a] Fei C, McLaughlin JK, Tarone RE, Olsen J (2007). Perfluorinated chemicals and fetal growth: a study within the Danish National Birth Cohort. Environ Health Perspect.

[b5-ehp0116-a0238a] Henderson WM, Smith MA (2007). Perfluorooctanoic acid and perfluorononanoic acid in fetal and neonatal mice following in utero exposure to 8-2 fluorotelomer alcohol. Toxicol Sci.

[b6-ehp0116-a0238a] Lederman SA, Paxton A, Heymsfield SB, Wang J, Thornton J, Pierson RN (1999). Maternal body fat and water during pregnancy: do they raise infant birth weight?. Am J Obstet Gynecol.

[b7-ehp0116-a0238a] Mardones-Santander F, Salazar G, Rosso P, Villarroel L (1998). Maternal body composition near term and birth weight. Obstet Gynecol.

[b8-ehp0116-a0238a] Sanin Aguirre LH, Reza-Lopez S, Levario-Carrillo M (2004). Relation between maternal body composition and birth weight. Biol Neonate.

[b9-ehp0116-a0238a] Sinclair E, Kim SK, Akinleye HB, Kannan K (2007). Quantitation of gas-phase perfluoroalkyl surfactants and fluorotelomer alcohols released from nonstick cookware and microwave popcorn bags. Environ Sci Technol.

[b10-ehp0116-a0238a] Tittlemier SA, Pepper K, Edwards L (2006). Concentrations of perfluorooctanesulfonamides in Canadian total diet study composite food samples collected between 1992 and 2004. J Agric Food Chem.

